# Targeted Approaches for In Situ Gut Microbiome Manipulation

**DOI:** 10.3390/genes9070351

**Published:** 2018-07-12

**Authors:** Hui Ling Lee, Haosheng Shen, In Young Hwang, Hua Ling, Wen Shan Yew, Yung Seng Lee, Matthew Wook Chang

**Affiliations:** 1 Department of Biochemistry, Yong Loo Lin School of Medicine, 8 Medical Drive, Singapore 117596, Singapore; bchlhl@nus.edu.sg (H.L.L.); bchhiy@nus.edu.sg (I.Y.H.); bchlingh@nus.edu.sg (H.L.); bchyws@nus.edu.sg (W.S.Y.); 2 NUS Synthetic Biology for Clinical and Technological Innovation (SynCTI), Life Sciences Institute, National University of Singapore, 28 Medical Drive, Singapore 117456, Singapore; hshen@u.nus.edu (H.S.); paeleeys@nus.edu.sg (Y.S.L.); 3 Department of Paediatrics, Yong Loo Lin School of Medicine, National University of Singapore, 5 Lower Kent Ridge Rd, Singapore 119074, Singapore; 4 Khoo Teck Puat-National University Children’s Medical Institute, National University Health System, 5 Lower Kent Ridge Rd, Singapore 119074, Singapore

**Keywords:** gut microbiome, microbiome modulation, prebiotics, probiotics, phage, CRISPR-Cas9

## Abstract

Microbial communities and their collective genomes form the gut microbiome, of which bacteria are the major contributor. Through their secreted metabolites, bacteria interact with the host, influencing human health and physiology. Perturbation of the microbiota and metabolome has been associated with various diseases and metabolic conditions. As knowledge on fundamental host-microbiome interactions and genetic engineering tools becomes readily available, targeted manipulation of the gut microbiome for therapeutic applications gains favourable attention. Manipulation of the gut microbiome can be achieved by altering the microbiota population and composition, or by modifying the functional metabolic activity of the microbiome to promote health and restore the microbiome balance. In this article, we review current works that demonstrate various strategies employed to manipulate the gut microbiome in situ to various degrees of precision.

## 1. Introduction

The human gastrointestinal tract (GIT) is colonised by trillions of microorganisms—namely bacteria, archaea, bacteriophages, and eukaryotes, which form a massive, ecological community through complex metabolic activities and constant host interactions. This community of microorganisms is defined as the gut microbiota, and its collective genomes are known as the gut microbiome [1,2]. Bacterial species are the most dominant members of the gut microbiota, and their genes account for approximately 99% of the gut microbiome [3]. Though the total bacterial cell mass in a healthy individual is estimated to be only around 200 g [4], the bacterial gut metagenome overwhelms the number of distinct genes found in the human genome by 150 times [5]. Thus, the gut microbiota has an extensive impact on both normal human physiology and disease susceptibilities, including defence against pathogens [6], nutrient utilisation [7], and peripheral education of the immune system [8].

The gut microbiome directs a host of metabolic pathways, interacting dynamically with the host through the metabolites generated. Changes in these metabolic pathways may alter the composition of gut lumen metabolites, perturbing the original balance in the host system. Responses from the host are dynamic and lead to the up- or down-regulation of related metabolic pathways until a new balance is attained. Dysbiosis of the microbiota in the host has been linked to various diseases, such as obesity, colorectal cancer, cardiovascular disease, and inflammatory bowel diseases (IBD) [9]. The restoration of healthy gut microbiota is believed to have positive effects on the treatment of these diseases. Moreover, the regulation of certain microbial groups in healthy individuals can be helpful in disease prevention. Therefore, manipulation of the gut microbiome presents valuable avenues for therapeutic and clinical applications.

**Figure 1:**
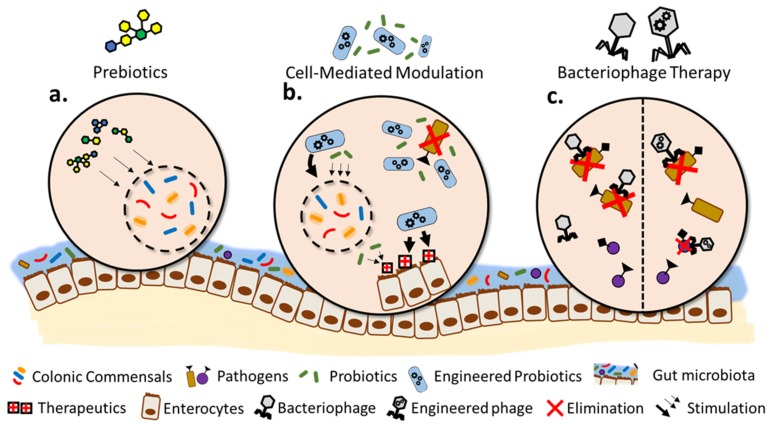
Overview of targeted methods to manipulate the gut microbiome. (**a**) Administration of targeted prebiotics to stimulate the growth of beneficial microbes; (**b**) use of targeted probiotics and engineered probiotics to eliminate pathogens or directly change the functional output of the gut microbiome; and (**c**) use of bacteriophages to eliminate specific species of pathogens or target pathogens with certain genes.

To positively impact health through therapeutic modulation of gut dysbiosis, the gut microbiome can be altered by changing the population, composition, and/or the functional output of metabolic pathways. Specifically, direct manipulation of the gut microbiome can be achieved by using targeted prebiotics, probiotics, engineered probiotics, or bacteriophages for targeted effects (Figure 1). Here, we give an overview of the progress in applying these approaches to directly modulate the gut microbiome in situ.

## 2. Prebiotics-Mediated Modulation of Gut Microbiome

Over the years, studies in the areas of metabolic syndrome, obesity, and prebiotics have significantly advanced knowledge on the gut microbiome and its role in maintaining human health. Modulating gut microbiota composition through diet manipulation, or, more specifically, through prebiotics, has garnered significant attention due to the (1) rising phenomenon of obesity in developed countries and increasing evidence of the gut microbiota’s influence, (2) ease of using prebiotics as a therapeutic approach, and (3) the increasing knowledge of the benefits of known metabolites through bacterial fermentation.

The current definition of prebiotics is proposed as ‘a substrate that is selectively utilised by host microorganisms conferring a health benefit’ [10]. Based on this, prebiotics need to be resistant to gastric digestion in the upper alimentary canal, and undergo fermentation by colonic bacteria to promote the growth of certain bacterial populations that are positively associated with health [10]. Due to these prerequisites, the most commonly known prebiotics used as target modifiers of the gut microbiome are non-digestible carbohydrates (NDC), such as fructo-oligosaccharides, galacto-oligosaccharides, inulin, and oligosaccharides [11]. Non-carbohydrate substrates that encompass similar characteristics, including polyphenols [12,13], phenolic acids [14], minerals, and peptides [15], are also being considered for use as prebiotics. However, the use of these non-carbohydrates is yet to be the focus in recent studies on prebiotics.

**Figure 2:**
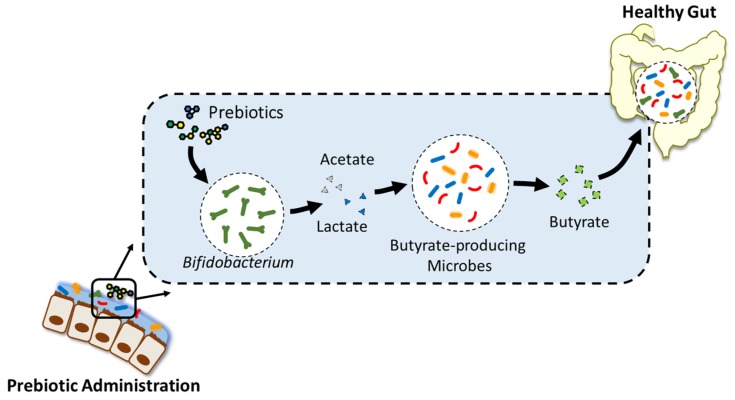
Cross-feeding effect between *Bifidobacterium* and butyrate-producing bacteria. *Bifidobacterium* utilises supplemented prebiotics, which stimulates their growth. Acetate produced by *Bifidobacterium* becomes a carbon source for butyrate-producing microbes, stimulating their growth and butyrate-producing activities and, in turn, modulating the microbiome function and improving gut health.

Though, seemingly, a general approach to manipulate the gut microbiome, recent studies on prebiotics focus on supplementing NDC to select *Bifidobacterium,* which is over-represented among the saccharolytic commensals [16], and is known to play important roles in carbohydrate utilisation [17]. Decreased levels of *Bifidobacterium* have been observed and associated with obesity and Type II diabetes, while supplementation of NDC in murine models [18] and human subjects [19,20] has been found to restore those levels. The major end-products of NDC fermentation are short-chain fatty acids (SCFAs), including acetate, propionate, and butyrate [21,22,23,24]. Acetate and propionate are involved in gluconeogenesis in hepatocytes and act as signalling molecules [25], while butyrate is an important carbon source for epithelial cells in the colon [26]. Interestingly, during NDC supplementation, the growth of butyrate-producing bacteria has been observed to increase, along with *Bifidobacterium* [18,27,28,29,30,31]. Prebiotic supplementation promotes the growth of *Bifidobacterium* and their utilisation of oligosaccharides to produce fermentative end products, mainly acetate and lactate. The excess acetate produced is subsequently utilised by butyrate-producing bacteria, such as *Faecalibacterium prausnitzii, Roseburia*, and *Eubacterium*, to produce butyrate. This ‘cross-feeding’ effect between *Bifidobacterium* and butyrate-producing bacteria ultimately leads to an increased butyrate production and augments beneficial effects to the host, such as improvement of the gut barrier integrity and pathogen inhibition (Figure 2) [26]. The health-promoting attributes of these butyrate-producing bacteria are supported in numerous diseased conditions, such as IBD [32], Crohn’s disease [33], and ulcerative colitis [34], where a significant reduction of butyrate-producing bacteria is reported. Though these butyrate-producing bacteria are not directly affected by the supplementation of oligosaccharides, their butyrate production is elevated due to the increased availability of fermentative end products generated by *Bifidobacterium*. Such cross-feeding behaviour reveals the limitations of prebiotics as targeted modifiers of the gut microbiota for specific effects. Nevertheless, this highlights the role of prebiotics in mediating complex interactions among populations in the gut microbiota, which presents opportunities to delve into more therapeutic approaches.

There are several limitations in the efficacy and efficiency of modulating gut microbiota via prebiotics. Efficacy can be compromised as a result of high inter-individual variability in the gut microbiome profile [35,36], in which individual responses may vary significantly, and results are difficult to reproduce. Efficiency, on the other hand, is diminished due to the transient lifespan of prebiotics as they pass through the GIT. While prebiotics favour and promote the growth of certain bacterial populations, this growth may be transient and may limit the extension of health benefits to the host. Prebiotics, therefore, must be constantly consumed or administered for a significant effect to be observed. Currently, natural prebiotics are most commonly used to selectively boost the growth of existing bacteria, or to modulate metabolic pathways present in the gut, though the precise mechanisms have yet to be elucidated.

## 3. Cell-Mediated Modulation of Gut Microbiome

### 3.1. Modulation through Targeted Probiotics

Beside prebiotics, probiotics are being used as targeted modifiers to modulate the gut microbiome. Probiotics are live microorganisms that, when administered in adequate amounts, confer health benefits to the host [37]. Probiotics generally promote gut health through mechanisms, such as regulation of pH levels and colonisation resistance [37]. Supplementation of probiotics is a direct way of manipulating the gut microbiome as it changes the microbiota composition. Most probiotics are, in fact, original gut commensals that have been isolated and characterised. Through various metabolic activities, probiotics can directly modulate the microbiota to exert beneficial effects on the host, as seen commonly during the treatment of gut dysbiosis. Well-known, gram-positive bacteria that confer such health benefits include *Bifidobacterium* and *Lactobacillus. Bifidobacterium adolescentis* IM38, for example, is reported to exert anti-inflammatory and anti-colitis effects on mice by modulating the ratio of Proteobacteria and Bacteroidetes populations [38]. *Bifidobacterium,* which has an innate metabolism for glycan harvesting, has also been shown to regulate and favour the expansion of species of its genus, such as *Bifidobacterium breve*, *Bifidobacterium longum,* and *Bifidobacterium bifidum*, leading to increased SCFAs, particularly acetate and butyrate, in the murine cecum [39]. Moreover, the supplementation of bifidobacterial strains was observed to modulate other populations of bacteria, such as *Lachnospiraceae*, which has been reported to be correlated with the early onset of diabetes [39,40]. Likewise, *Lactobacillus* strains, such as *Lactobacillus rhamnosus* GG and *Lactobacillus sakei*, have been shown to regulate gut microbiota profiles and lower obesity-related markers in mice, presumably through antagonistic activities against *Clostridium* [41]. In addition to gram-positive bacteria, gram-negative probiotics, such as *Escherichia coli* Nissle 1971 (EcN), have also been well-characterised [42]. Particularly, EcN administration has been shown to inhibit gut pathogens through various mechanisms, such as nutrient limitation [43] and colonisation resistance [44,45]. These examples demonstrate that the administration of targeted probiotics can directly modulate the gut microbiota by altering the population and metabolic output of the microbiome to actively re-establish a healthy balance. The beneficial effects of probiotics can be further harnessed through concurrent supplementation of both prebiotics and probiotics, also known as synbiotics, where the growth of the supplemented probiotics is specifically enhanced by the prebiotics [46,47]. In synbiotics, both prebiotics and probiotics are the modifiers of the microbiome. Specifically, the main role of prebiotics in synbiotics is to enhance the growth of probiotics so that the sheer increase in the population would lead to beneficial effects.

Despite these promising proofs-of-concept studies, probiotics have not shown significance in many clinical trials [48,49]. This is likely due to differences in genetic backgrounds and gut microbiome profiles across individuals [50,51]. One of the major drawbacks lies in the difficulty of isolating and culturing intestinal commensal microbes, which are mostly obligate anaerobes [52]. Furthermore, probiotics are known to colonise the intestinal tract for only short durations, which explains the frequent administration required for tangible health effects [53]. To address these drawbacks, researchers are seeking new methods to isolate and culture gut commensals [54,55,56]. The findings from these studies can contribute to discovering potential probiotic strains with distinct health effects and a niche colonisation.

### 3.2. Targeted Modulation through Engineered Probiotics

Probiotics can directly alter the composition of the gut microbiota for host benefits, yet the precise mechanisms are known for only a few strains [37]. As such, targeted approaches to modulate the gut microbiome are more likely to achieve therapeutic significance for the host.

One targeted approach is through the rational engineering of probiotics to directly alter the functional output of the microbiome; a specific metabolic output is achieved and host benefits are conferred without unnecessarily causing major alterations to the microbiota population and composition. This approach often involves introducing non-native pathways in probiotics through heterologous expression of enzymes. The additional functionalities allow probiotics to wield more targeted effects on the host, rather than confer general positive host benefits. For example, EcN was engineered to produce *N*-acylphosphatidylethanolamines, which are precursors to a family of anorexigenic lipids [57]. Incorporation of this engineered EcN into mice on a high-fat diet showed a reduction in fat mass and weight gain compared to mice supplemented with wild-type EcN [57]. Significant changes in fatty acid oxidation-related gene expression were observed, demonstrating the targeted effect of an engineered microbe [57]. In another study, EcN was engineered as a chassis to deliver glucagon-like peptide-1 (GLP-1) in diabetic rats to restore the insulin sensitivity of intestinal cells [58]. This approach directly addresses the impaired function of the gut microbiome as it has been postulated that diminished SCFAs levels in the diabetic gut with dysbiosis are linked to reduced GLP-1 secretion [59]. Other studies have harnessed lactic acid bacteria, specifically *Lactococcus lactis*, for the production and delivery of bioactive molecules, such as interleukin-10, for specific metabolic effects, including anti-inflammatory effects [60,61,62]. Apart from the delivery of therapeutic compounds, probiotics were also engineered for pathogen sensing, suppression, and elimination, specifically in the gut. For example, an improved ‘sense-kill’ system [63] employed in EcN was shown to successfully eradicate and prevent *Pseudomonas aeruginosa* colonisation in both *Caenorhabditis elegans* and murine models [64]. In a recent study, Mao et al. [65] engineered lactic-acid producing *L. lactis* to detect quorum-sensing molecules produced by *Vibrio cholera*, and express an enzymatic reporter upon detection. The engineered *L. lactis* was found to specifically detect and report the presence of *V. cholerae* and significantly improve the survival rate of infected mice [65]. Besides well-known probiotics, there has been recent progress in the engineering of gut commensals, which also presents great potential for bio-medical applications. Specifically, in *Bacteroides thetaiotaomicron,* components for tunable gene expression were developed and characterised. Expected functional outputs were observed in mice after administration of these engineered *B. thetaiotaomicron* [66,67]. These present opportunities to harness such engineered commensals for therapeutic purposes. Taken together, these studies demonstrate the feasibility of using engineered bacteria to directly manipulate the functional output of the microbiota without major modulation of the microbiota population and composition.

Precision approaches that integrate prebiotics and engineered probiotics to accentuate the targeted effects have also emerged. EcN has been reported to be engineered to specifically target and kill colorectal cancer cells in vivo [68]. Interestingly, the engineered EcN expresses and secretes a myrosinase that transforms host-ingested glucosinolate, a compound mostly found in cruciferous vegetables, into the anti-carcinogenic compound, sulphoraphane [68]. This study demonstrates how components in the normal diet (possibly emerging prebiotics) and engineered probiotics can be harnessed simultaneously to render a targeted effect on the host through modulating the functional output of the microbiome.

## 4. Phage-Mediated Modulation of Gut Microbiome

While the use of probiotics to promote or compete with natural resident bacteria of the gut is actively being explored, the use of genetic tools to selectively remove a targeted population is also being investigated. The discovery of bacteriophages in the earlier advent of phage therapy [69], combined with a more recent discovery of the clustered regularly interspaced short palindromic repeats (CRISPR) and the CRISPR-associated nuclease 9 (Cas9) system, has led to an array of strategies to manipulate the gut microbiome with precision [70,71]. For instance, engineered phage (with the CRISPR-Cas9 system) can be employed to target pathogenic bacteria, or remove the population of bacteria that aids pathogenic bacterial growth, thereby fine-tuning and restoring the balance of the gut microbiome. In recent years, several groups have attempted to show the effectiveness of using bacteriophages to eliminate specific populations of bacteria without disrupting other commensals. For example, virulent bacteriophages were tested against adherent invasive *Escherichia coli* (AIEC) found on the mucosa of the ileum of patients with Crohn’s disease. A considerable reduction of AIEC was observed after phage treatment in vivo, demonstrating a microbiota composition change through pathogen removal [72]. Similarly, the effectiveness of the oral administration of a bacteriophage cocktail showed that phage treatment yielded greater clearance of the pathogen compared to the use of ampicillin in treating *Shigella*-challenged mice [73]. The treatment was also specific in removing the target population without affecting other commensals [73]. Other studies have demonstrated the specificity of bacteriophages in altering the microbiota composition through pathogen removal and reduction, without perturbation to other commensal populations, include the targeting of *Enterococcus faecalis* [74], *Listeria monocytogenes* [75], and *Clostridium difficile* [76].

While phage therapy is used for the population removal of pathogens, CRISPR/Cas9 can be used to manipulate and differentiate genetically heterogeneous bacteria, even of the same species. Currently, one of the pressing clinical issues is antibiotic resistance and the rise of multi-drug resistant (MDR) bacteria that can result in superinfections [69]. Unlike conventional drugs, the CRISPR/Cas9 system targets specific bacteria at the gene level to selectively remove pathogens, making this tool a potential antimicrobial adjuvant to improve antibiotic treatment. This concept was demonstrated by Citorik et al. [77], where CRISPR/Cas9 was delivered using bacteriophages, targeting the *ndm-1* gene, which codes for the broad-spectrum carbapenemase, New-Delhi metallo-β-lactamase. *Ndm-1* targeting CRISPR/Cas9 specifically eliminated *E. coli* harbouring the gene without affecting wild-type, or other, *E. coli* strains present in a synthetic consortium of microbes [77]. Another notable example is the re-sensitisation of bacteria to antibiotics and immunisation of bacteria to incoming plasmids conferring antibiotic resistance using temperate phages. Specifically, Yosef et al. used CRISPR/Cas9 to target *ndm-1* and *ctx-M-15*, which expresses a broad-spectrum beta-lactamase, and effectively selected the transduced bacteria that were antibiotic-sensitive [78]. Though these studies are still mostly in vitro, they pave the way to advance future work using CRISPR/Cas9 to manipulate the gut microbiome by discriminating at the gene level to change the characteristics and functional output of the gut microbiome for therapeutic applications.

While phage therapy and the CRISPR/Cas9 system hold promise for future applications, there are several concerns for each of them. One of the concerns for phage therapy is the unintended activation of host immune responses. Phage therapy relies on the lysis of the bacterial host, which releases unwanted cellular components, such as endotoxins, into the gut lumen environment that may trigger an immune response [79]. Though the probability of immune activation by phages cannot be eliminated, clinical trials using phage therapy has thus far shown to be well-tolerated without any adverse side effects. Interestingly, a study demonstrated that the use of carbapenems caused more endotoxins than phages [80]; emerging alternatives to circumvent this issue include engineering lysis-deficient phages [81]. For CRISPR/Cas9, some concerns, including off-target gene targeting and escape rates, are summarised in a review, along with recent works [77,82,83]. These concerns can be addressed progressively as areas in CRISPR/Cas9 research, such as optimising gene multiplexing and continuing the development of increased specificity and efficiency of CRISPR/Cas9 in target organisms. As phage therapy and the CRISPR/Cas9 system gain momentum as tools for microbiome manipulation, more in vivo work and clinical experimentation are needed to ascertain the various effects of using such viral-based approaches.

## 5. Conclusions and Future Outlook

In this review, we focused on recent work showing how the gut microbiome can be directly manipulated in a targeted manner in situ. Population modulation through composition alteration can be achieved though the administration of targeted modifiers, such as prebiotics, probiotics, and phages, with measurable improvements in health markers for various gut disorders and metabolic conditions. Targeted manipulation of known pathways in the gut microbiome can also be achieved to alter the functional metabolic output of the microbiome. Manipulating the gut microbiome fundamentally relies on the extent of knowledge about the microbiota, the multitude of metabolic pathways present in the GIT, and the complex interactions with the host. As the therapeutic applications of gut microbiome manipulation are still in the incipient phase, current efforts to advance the fundamental understanding of the gut microbiome and its host interactions should be fortified and strongly encouraged to actualise its therapeutic and clinical potential.
